# Acantholytic Acanthoma Arising in Severely Photodamaged Skin: A Case Report

**DOI:** 10.7759/cureus.97539

**Published:** 2025-11-23

**Authors:** Beyza Nur Elibol, Funda Erduran, Huban Sibel Orhun

**Affiliations:** 1 Dermatology, Ankara City Hospital, Ankara, TUR; 2 Pathology, Ankara City Hospital, Ankara, TUR

**Keywords:** acantholytic acanthoma, actinic damage, benign epidermal tumor, dermatopathology, facial lesion, photodamaged skin, ultraviolet radiation

## Abstract

Acantholytic acanthoma is a rare benign epidermal tumor that usually occurs in elderly individuals as a solitary, asymptomatic, keratotic papule, most commonly located on the trunk. Histopathologically, it is characterized by hyperkeratosis, papillomatosis, acanthosis, and acantholysis at various levels of the epidermis. Although its exact etiology remains unclear, it has been suggested that acantholytic acanthoma may arise in association with immunosuppressive states or chronic UV-induced skin damage. We present a rare case of acantholytic acanthoma that developed on the face of a 73-year-old male patient with severely photodamaged skin and a history of multiple cutaneous malignancies. The patient presented with an asymptomatic, erythematous, 1.5 cm papule on the right cheek. His medical history included spindle-cell squamous cell carcinoma of the right auricular helix and infiltrative-type basal cell carcinoma of the right dorsal forearm. Dermoscopic examination of the lesion revealed white dots and telangiectasias on an erythematous background. Histopathologic evaluation of the punch biopsy specimen showed marked hyperkeratosis, papillomatosis, acanthosis, and intraepidermal acantholysis, findings consistent with acantholytic acanthoma. Complete surgical excision was performed, and no recurrence was observed during follow-up. Acantholytic acanthoma is most often found on the trunk, and facial localization is extremely uncommon. In our case, the lesion’s occurrence in a patient with severe photodamage and a prior history of different skin cancers is noteworthy and may suggest a possible relationship between UV exposure and the development of acantholytic acanthoma. Histopathologically, it must be differentiated from other acantholytic dermatoses such as pemphigus vulgaris, Hailey-Hailey disease, and Grover’s disease, as well as from acantholytic actinic keratosis and basal cell carcinoma. Acantholytic acanthoma is distinguished by its solitary presentation, benign clinical course, and absence of cellular atypia or dysplasia. Complete excision is curative, and recurrence is rare. This case contributes to the expanding clinical spectrum of acantholytic acanthoma by highlighting that it can develop on severely photodamaged skin. Moreover, it underscores the importance of considering acantholytic acanthoma in the differential diagnosis of solitary erythematous papular lesions on the face, especially in elderly individuals with chronic sun damage. Early recognition and appropriate management can ensure excellent outcomes and prevent unnecessary interventions.

## Introduction

Acantholytic acanthoma is a rare benign epidermal tumor that typically presents in elderly individuals as a solitary, asymptomatic, keratotic papule, most commonly located on the trunk [1]. Histopathologically, it is characterized by hyperkeratosis, acanthosis, and papillomatosis, accompanied by acantholysis at various levels of the epidermis [2]. Although the exact etiologic factor remains unclear, an increased incidence has been reported in patients who have undergone renal transplantation, suggesting a possible association with immunosuppression [3]. The lesion is generally considered a distinct clinicopathologic entity, although debate exists regarding whether it represents a variant of seborrheic keratosis, actinic keratosis, or a reactive epidermal proliferation secondary to chronic irritation or UV exposure [4]. Additionally, chronic UV-induced damage to epidermal desmosomes has been proposed as a potential contributing mechanism in sun-exposed skin.

Most cases described in the literature are located on the trunk, and facial involvement is uncommon, making such presentations clinically significant. In this case report, we present a rare example of acantholytic acanthoma located on the face, an unusual site, occurring on severely photodamaged skin in a patient with a history of spindle-cell squamous cell carcinoma and basal cell carcinoma.

## Case presentation

A 73-year-old male patient presented to our dermatology outpatient clinic with a slowly enlarging, asymptomatic papule on the right cheek. The patient reported that the lesion had been present for several months and had gradually increased in size without associated pain, pruritus, or bleeding. His medical history included spindle-cell squamous cell carcinoma of the right auricular helix, diagnosed in December 2021, and infiltrative-type basal cell carcinoma of the right dorsal forearm, diagnosed in January 2022. He had Fitzpatrick skin type II and exhibited pronounced chronic actinic skin damage due to long-term sun exposure.

**Figure 1:**
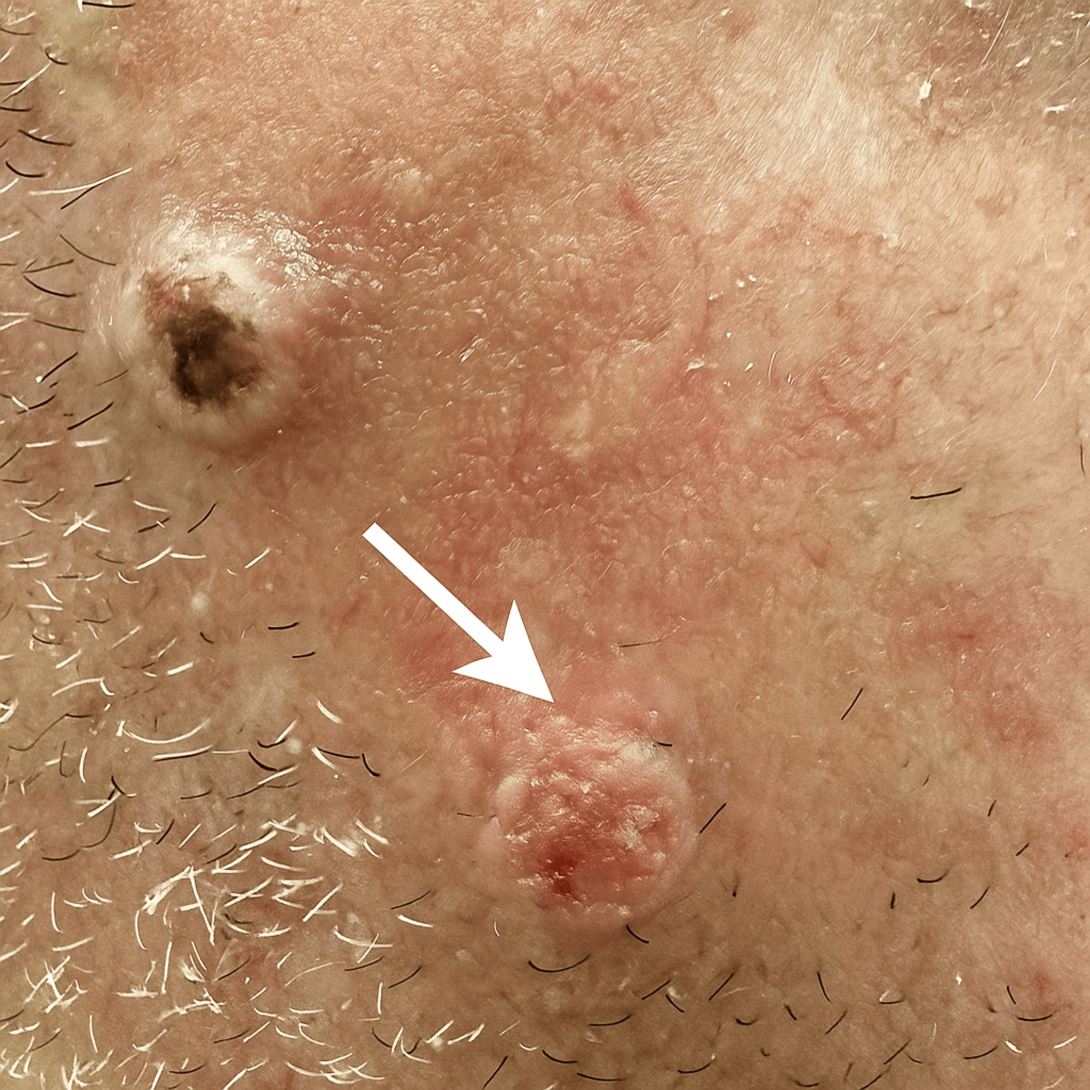
Asymptomatic erythematous papule with telangiectasias on the right cheek

On physical examination, a well-demarcated, erythematous papule measuring approximately 1.5 cm in diameter was noted on the right malar region (Figure 1). The surface of the lesion appeared keratotic and slightly irregular. Multiple actinic keratoses were also observed on the frontal scalp and both auricular helices. No regional lymphadenopathy was detected.

**Figure 2:**
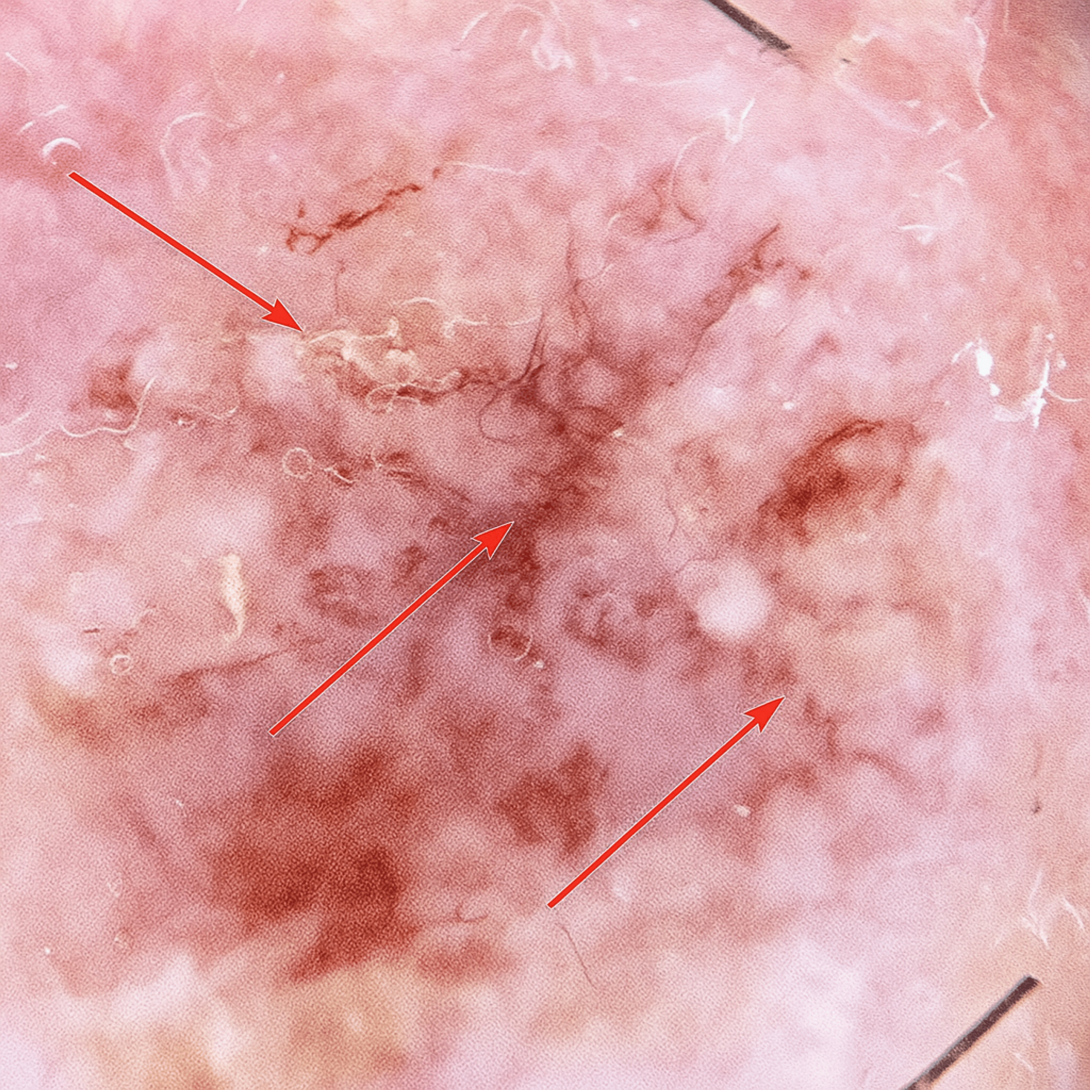
Dermoscopic appearance of the lesion

Dermoscopy of the cheek lesion demonstrated white structureless areas, focal scale, and fine telangiectatic vessels distributed over an erythematous background, without features suggestive of basal cell carcinoma or squamous cell carcinoma (Figure 2). The dermoscopic image was obtained during a scheduled follow-up evaluation.

**Figure 3:**
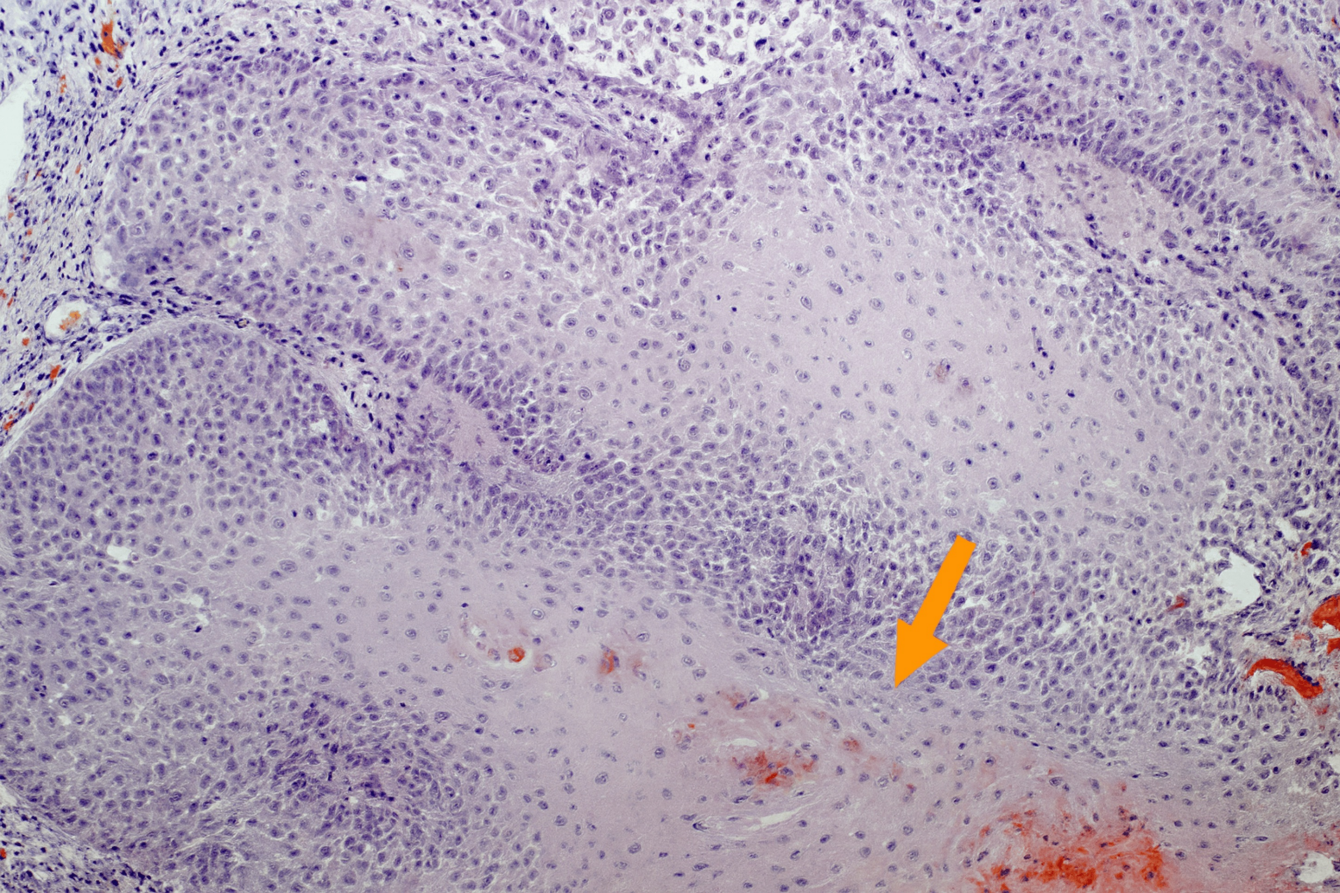
Intraepidermal lesion showing acantholysis (H&E X100)

Given the patient's history of multiple cutaneous malignancies and the atypical appearance of the lesion, a punch biopsy was performed for diagnostic clarification. Histopathologic evaluation revealed marked hyperkeratosis, papillomatosis, acanthosis, and prominent intraepidermal acantholysis involving multiple levels of the epidermis, consistent with acantholytic acanthoma (Figure 3). No cytologic atypia or dysplasia was identified.

The patient was informed about the benign nature of the lesion, and complete excision was planned for definitive treatment. He remained clinically stable during follow-up.

## Discussion

Acantholytic acanthoma is a benign epidermal tumor that typically presents as a solitary hyperkeratotic papule, most often on the trunk of elderly individuals [1]. Although its exact etiology remains uncertain, several mechanisms have been proposed, including chronic UV damage and localized desmosomal disruption, which may contribute to acantholysis within the epidermis. Some reports have also described acantholytic acanthoma in immunosuppressed individuals, suggesting that impaired immune surveillance may play a role in its development [3]. Despite these hypotheses, the lesion continues to be regarded as a distinct clinicopathologic entity rather than a variant of seborrheic keratosis or actinic keratosis [4], and it is included in the current WHO Classification of Skin Tumours as a benign epidermal neoplasm [5].

Histopathologic evaluation remains essential for a definitive diagnosis. The characteristic features, hyperkeratosis, papillomatosis, acanthosis, and multifocal acantholysis, may overlap with other acantholytic dermatoses such as pemphigus vulgaris, pemphigus foliaceus, Hailey-Hailey disease, and Grover’s disease. However, these inflammatory disorders typically present with widespread, symptomatic eruptions rather than a solitary, asymptomatic lesion, which helps distinguish them clinically [2]. In our case, the combination of dermoscopic findings, white structureless areas, and telangiectatic vessels on an erythematous background, and histopathologic evidence of acantholysis supported the diagnosis.

The differential diagnosis includes verrucous dyskeratoma, acantholytic actinic keratosis, and basal cell carcinoma. Verrucous dyskeratoma is characterized by acantholysis with prominent dyskeratosis involving a follicular unit, while acantholytic actinic keratosis typically demonstrates cytologic atypia and occurs exclusively on chronically sun-damaged skin. Basal cell carcinoma may mimic acantholytic acanthoma clinically, especially in the head and neck region, where pearly papules with telangiectasias are common [3]. Given that our patient had a history of both spindle-cell squamous cell carcinoma and basal cell carcinoma, histologic confirmation was critical to avoid misdiagnosis and unnecessary overtreatment.

Facial involvement is uncommon, as most reported acantholytic acanthomas occur on the trunk [4]. The unusual location in our case, on severely photodamaged facial skin, raises the possibility that chronic ultraviolet exposure may contribute to lesion development in susceptible individuals. Additionally, the patient’s history of multiple nonmelanoma skin cancers may reflect cumulative ultraviolet injury or altered epidermal integrity, potentially facilitating focal acantholysis.

Management is straightforward, as complete surgical excision is considered curative and recurrence is rare [1,2]. Nevertheless, awareness of this entity is important, particularly when it occurs in atypical locations or in patients with significant actinic damage, as misinterpretation of dermoscopic or clinical features may raise suspicion for malignancy. Accurate clinicopathologic correlation ensures appropriate diagnosis and prevents unnecessary aggressive treatment.

## Conclusions

This case highlights an uncommon facial presentation of acantholytic acanthoma, a lesion that typically arises on the trunk of elderly individuals. The patient’s history of significant photodamage underlines the importance of careful evaluation of solitary facial papules, particularly in older adults. Recognition of the characteristic clinical and histopathologic features is essential for distinguishing this benign entity from other acantholytic or malignant lesions. Early and accurate diagnosis helps prevent unnecessary interventions and ensures appropriate management.
